# Parenting Practices and Adolescents’ Eating Behaviors in African American Families

**DOI:** 10.3390/ijerph19010110

**Published:** 2021-12-23

**Authors:** Lillie Monroe-Lord, Blake L. Jones, Rickelle Richards, Marla Reicks, Carolyn Gunther, Jinan Banna, Glade L. Topham, Alex Anderson, Karina R. Lora, Siew Sun Wong, Miriam Ballejos, Laura Hopkins, Azam Ardakani

**Affiliations:** 1 Center for Nutrition, Diet and Health, University of the District of Columbia, Washington, DC 20008, USA; 2 Department of Psychology, Brigham Young University, Provo, UT 84602, USA; blake.jones@byu.edu; 3 Department of Nutrition, Dietetics & Food Science, Brigham Young University, Provo, UT 84602, USA; rickelle_richards@byu.edu; 4 Department of Food Science and Nutrition, University of Minnesota, St. Paul, MN 55108, USA; mreicks@umn.edu; 5 Human Nutrition Program, Department of Human Sciences, Ohio State University, Columbus, OH 43210, USA; Gunther.22@osu.edu; 6 Department of Human Nutrition, Food and Animal Sciences, University of Hawaii-Manoa, Honolulu, HI 96822, USA; jcbanna@hawaii.edu; 7 Department of Applied Human Sciences, Kansas State University, Manhattan, KS 66506, USA; gtopham@ksu.edu; 8 Department of Nutritional Sciences, University of Georgia, Athens, GA 30602, USA; fianko@uga.edu; 9 Department of Exercise and Nutrition Sciences, The George Washington University, Washington, DC 20052, USA; klora@email.gwu.edu; 10 School of Biological and Population Health Sciences, Oregon State University, Corvallis, OR 97331, USA; siewsun.wong@oregonstate.edu; 11 Nutrition & Exercise Physiology, Washington State University, 412 E. Spokane Falls Blvd, Spokane, WA 99202, USA; miriam.s.ballejos.civ@mail.mil; 12 Department of Public Health and Prevention Sciences, Baldwin Wallace University, 275 Eastland Rd., Berea, OH 44017, USA; lhopkins@bw.edu; 13 Department of Nutritional Sciences, Howard University, Washington, DC 20059, USA; azam.ardakani@bison.howard.edu

**Keywords:** African American, eating behavior, parenting practice, adolescents, low income

## Abstract

Parents play an important role in developing the eating behaviors of their children by adopting specific parenting practices. As the prevalence of obesity is high amongst African American adolescents, investigations into associations of specific parenting practices and adolescents’ eating behaviors are essential. In this exploratory study, 14 African American parent–adolescent dyads were interviewed to characterize the influence of eight different parenting practices on the consumption of three main food categories (dairy, fruits and vegetables, and unhealthy snacks). The results revealed that authoritarian parenting practices were correlated with a higher BMI percentile in adolescents, whereas modeling and monitoring are correlated with a higher parent BMI. In addition, reasoning, monitoring, modeling, and authoritative parenting practices were associated with less unhealthy snack consumption among adolescents. Reasoning and monitoring were the only parenting practices associated with higher fruit and vegetable consumption. Finally, a significant correlation was found between eating fruits and vegetables and unhealthy snacks and the location of eating. In conclusion, different parenting practices and environmental factors may impact BMI and food consumption of African American dyads. The results of this study can be used to guide improvement in, and/or development of, nutritional education interventions considering the cultural differences of racial minorities.

## 1. Introduction

Childhood obesity is an increasingly common health condition. According to the Centers for Disease Control and Prevention (CDC), the prevalence of obesity amongst youth (12–19 years) in the United States is approximately 21% based on an estimated Body Mass Index-for-age (BMI-for-age) at or above the 95th percentile [1]. The National Center for Health Statistics estimated that 24% of African American youth have obesity [1]. It was also estimated that 80% of overweight and obese adolescents will continue this condition into adulthood [2,3]. Obese adolescents are at higher risk of developing chronic diseases such as diabetes and cardiovascular diseases in adulthood [4,5]. Unsuitable dietary habits, lack of physical activity, and genetic predisposition are the leading causes of the increased obesity rate [6,7].

Social cognitive theory (SCT) suggests that health behaviors are influenced by personal behaviors and experiences and the actions of others, in addition to environmental factors [8]. This theory has been used many times to identify adolescent eating behaviors, because it can identify meaningful links between eating behaviors and parenting practices. Obesogenic eating behaviors among adolescents can occur during both family eating occasions and independent eating occasions (IEOs). Thus, SCT—as a theoretical framework—considers self-efficacy, which is a vital, pre-conditional element for health behavior. Self-efficacy, resulting in self-regulation, leads to long-term and continuous positive behavior self-maintenance [9].

Several studies have found the home environment to be an effective element for the improvement in the healthy eating behaviors of adolescents [10,11,12]. The home environment can be categorized by parenting practices, family meals, having disciplined eating patterns, availability of food and drinks, watching television, or playing during a meal. Managing these elements wisely not only prevents obesity in adolescents, but can also promote healthy eating behaviors in adolescents [12,13,14].

Adolescence is a period in which individuals seek autonomy in their behavior. Parenting practices can help adolescents adopt healthier eating habits [15]. Newman et al. revealed that authoritative parenting practices (empathy, moderate number and consistency of rules, and supportive behavior) result in a healthier eating pattern compared to permissive parenting practices (empathy, indulgence, lenience, and few rules) [16]. The primary way that parents can impact healthy eating behaviors during IEOs is by controlling the availability and accessibility of healthy and unhealthy foods and beverages at home [17]. The accessibility and availability of fruits and vegetables and decreased access to sweetened beverages was related to healthier eating habits in adolescents [18]. Parenting practices that encourage the home availability of calcium-rich foods and setting rules and expectations for this type of food lead to their higher consumption [19]. Setting rules and expectations can encourage the control of caloric intake and portion size, along with establishing a healthy eating pattern. The existence of at least one food-related rule at home results in selecting healthier foods during IEOs [20]. However, the type of rules and expectations has an important influence on an adolescent’s healthy eating patterns. Wang and Fielding revealed that having healthy and positive rules at home increases the selection of healthier foods when adolescents are alone without supervision [20]. Conversely, children who receive controlling parenting practices are limited in their ability to regulate their food consumption [21]. Some parents also use specific foods, usually sweet or salty snacks, as a reward for desirable behavior. As this parenting practice can increase the adoption of unhealthy eating patterns in children, it does not lead to enforcement of the desired behaviors [22].

Adoption of parenting practices is dependent on culture. Understanding the cultural background of families can assist in the development of influential intervention strategies to address obesity and the adoption of healthy eating patterns [22,23]. A study by Sherry et al. showed that the majority of the African American population adopts authoritarian parenting practices (rigid, strict, and controlling) [24]. These parenting practices can sometimes evoke a sense of safety and can be protective among adolescents, but can prevent the development of their self-efficacy [25,26]. Other studies have shown that adolescents who experience strict and controlling parenting practices have unhealthy eating habits and consume more sugar-sweetened beverages [21,27].

The primary objective of this exploratory study was to identify the parenting practices of African American parents associated with healthy food choices of their child (aged 10−13 years) based on parent and child in-depth interviews and surveys. The secondary objective was to examine associations among other environmental factors and consumption of different food items. These environmental factors include the location of eating occasions, just eating vs. engaging in other activities during eating occasions, and eating alone or eating with friends and family. The findings of this study can be used to design a culturally appropriate nutrition education intervention to help African American parents adopt the best parenting practices to promote better eating habits.

## 2. Materials and Methods

### 2.1. Participants

In this study, we used in-depth interviews of parent–child dyads as part of a multi-state project that identified parenting practices and their influence on eating behavior. Interviews were completed for 51 early adolescents aged 10–13 years and their parents, including 14 African Americans used for this study. They were from five states (Georgia, Hawaii, Minnesota, Ohio, and Connecticut) and the District of Columbia. The inclusion criteria for parents included ability to read and speak English, live with and be responsible for providing food for adolescents aged 10–13 years, and identify as a low-income household. Based on the 2016 United States Poverty Guidelines, a household at or below 185% of the federal poverty level was eligible for some federal nutrition assistance programs and therefore was considered low-income in regard to the inclusion criteria for this study [28].

### 2.2. Procedure

The protocol of this study was approved by the Institutional Review Boards of the institutions involved in the data collection. All participants (both parents and adolescents) signed an informed consent form prior to participation in the study. Adolescent participants met with the research team twice. In the first session, a consent form was completed, the study protocol was explained, and instruction was provided to adolescents on how to collect food photos. A diary of each food item eaten during the day was recorded through food photography. The second interview session was based on the food photos with questions that probed about each food consumed and the context around consumption. Interviews were conducted in person and parents and adolescents were interviewed separately. All interviews were audio-recorded. Interviews for both the parents and children lasted approximately 60 min. Parents also completed a survey as previously reported [17,29,30,31], including a total of 68 questions about the frequency of parenting practices thought to influence dietary intake of adolescents. Responses to the questions were reported in a five-point Likert scale ranging from ‘strongly disagree’ to ‘strongly agree’ or ‘never’ to ‘always’ [17]. The parents’ and adolescents’ height, weight, and waist circumference were measured in duplicates following standard protocols [32] by trained research assistants. Body mass index (BMI) and BMI-for-age percentiles were calculated for parents and adolescents, respectively.

### 2.3. Adolescent Interviews

The food item consumption of the adolescents in this study was retrieved from interviews and then coded according to food categories including dairy, fruit, and vegetables, and unhealthy snacks. The details of each food category are listed in Table 1. Environmental variables such as adolescents’ location of eating (i.e., home, school, car/bus, or other), activity while eating, and accompanying individuals during eating were considered in this study.

### 2.4. Data Analysis

SAS software version 9.4 (SAS Institute, Cary, NC, USA) was used to conduct the statistical analysis for this study. Spearman’s rank correlation was used to test the association of the BMI of parent and adolescent dyads with their parenting practices. Wilcoxon’s rank sum tests were used to test for the association of parenting practices, environmental factors, and adolescent and parent perceptions of parenting practices with the consumption of different food categories during adolescents’ eating occasions (EOs). EOs included both IEOs and non-IEOs. For this paper, frequency of all EOs were considered for evaluating overall eating behaviors. For each food category, we summed the number of items by eating occasion. Similarly, we summed up food items by eating location to examine consumption by location. An exploratory factor analysis with a varimax rotation was used to identify parenting practice types based on responses to the parent survey. It was run on the dataset from the full study (*n* = 51) [33].

## 3. Results

### 3.1. Parenting Practices

An initial factor analysis was run for a set of 35 items. This analysis produced four factors, and one item was excluded because it did not load with any of the factors. A second, similar factor analysis was run on a separate group of 33 items. This analysis also produced four factors, and two items were excluded from the final factors. The total of 68 items are listed in Table 2. The factor loadings and variance for each factor are shown in Table 3. A total of eight parenting practice factors were identified through the factor analysis. The parents received a score on all eight parenting practices. Among the interviewed African American families, the parents agreed most strongly with rule-setting and authoritarian practices. In contrast, the parents tended to be neutral about or disagree with neglecting practices.

### 3.2. Demographic Data

The study sample consisted of 14 African American dyads of parents and adolescents who were interviewed. The dyads answered questions about parenting practices, home environment, and food item consumption. The sample characteristics are presented in Table 4. The study population was composed of six boys (43%) and eight girls (57%); most were 10 years old (86%). Most of the parents were mothers (79%), and 71% had some college education or above. The household income for all participants was less than USD 45,000 annually. In addition, 86% of adolescents lived in single-parent households (single, divorced, or separated).

### 3.3. Demographic and Parenting Practices

The relationships between parenting practices and adolescent sex, parent sex, education, income, and marital status were examined. Authoritative parenting practice showed a marginally significant difference (*p* = 0.06) by adolescent sex, with a higher authoritative score among parents of boys. The copying parenting practice showed a marginally significant difference (*p* = 0.05) by parent sex, with a higher copying score for women parents. The parents with higher education scored higher on reasoning practice (*p* = 0.05). No other relationships were found between parenting practices and the demographic variables.

### 3.4. BMI and Parenting Practices

The adolescents had a mean (SD) BMI percentile of 69.0 (33.4) with a median of 85 (*n* = 13). The parents had a mean (SD) BMI of 32.7 (9.5) and a median BMI of 29.1 (*n* = 8). Table 5 describes the correlation between the BMI of both parents and adolescents with each of the parenting practices. We found a marginally significant relationship between authoritarian parenting practices and adolescent BMI percentile (*p* = 0.08), suggesting that a more authoritarian style may lead to a higher BMI percentile in adolescents. Among the parents, a higher BMI was significantly associated with a higher modeling score (*p* = 0.003) and marginally associated with a higher monitoring score (*p* = 0.07).

### 3.5. Food Consumption and Parenting Practices

The association between parenting practice and the consumption of the three food categories (dairy, fruits and vegetables, and unhealthy snacks) was examined (Table 6). There were no associations between the consumption of dairy items and parenting practices. Higher reasoning and monitoring scores were associated with a higher consumption of fruits and vegetables among adolescents (*p* = 0.02 and *p* = 0.02, respectively). The consumption of unhealthy snacks was negatively associated with reasoning, monitoring, modeling, and authoritative parenting practices (*p* = 0.006, *p* = 0.003, *p* = 0.04, and *p* = 0.03, respectively). Higher scores on all four of these parenting practices was associated with a lower consumption of unhealthy snacks. Interestingly, higher scores for reasoning and monitoring parenting practices were associated with higher consumption of fruit and vegetables and lower consumption of unhealthy snacks.

### 3.6. Environmental Factors and Parenting Practices

Environmental factors associated with the consumption of different food categories—including eating location, engaging in another activity while eating or just eating, and eating alone or with someone else—were examined here. The detailed consumption of each food category is reported in Table 7. A total of 93 EOs were reported by 14 African American adolescents, with dairy consumed on 23% of the EOs, fruits and vegetables on 38% of the EOs, and unhealthy snacks on 39% of the EOs. The majority of the EOs occurred at home (61.3%) or at school (20.4%). More than half of the EOs (63.4%) occurred while engaged in other activities, and 81.7% of the EOs were consumed with someone else. The EO location was significantly associated with the consumption of fruits and vegetables and unhealthy snacks (*p* = 0.01 and *p* = 0.05, respectively). Interestingly, among the 19 EOs in school, 13 (68%) included fruits and vegetables. In contrast, fruits and vegetables were consumed on only 33% of EOs at home. Among the eight EOs in a vehicle, 75% included unhealthy snacks.

## 4. Discussion

In this study, we evaluated the relationship between different parenting practices and the eating behaviors and obesity of African American adolescents. Studies evaluating the parenting practices of African American parents and their influence on adolescents’ healthy eating behaviors are scarce. The results of this study indicate that amongst the sample of low-income African American families, parents applying more authoritarian approaches had adolescents with higher BMI percentiles. The literature shows that authoritarian parenting practices, which are characterized by rigid, strict, and controlling practices, are common among African American parents [25]. Our results also show an association between authoritarian parenting practice and higher BMI-percentile amongst African American adolescents. This association is important finding as gives rise to the importance of health policy in reducing obesity. Authoritarian parenting practices create an obstacle for the growth of self-efficacy in terms of adolescents’ healthy eating habits and healthy weight status [27]. This finding is also consistent with the study conducted by Berge et al., who found authoritarian practices to be a predictor for higher BMI in adolescent boys [34]. No other parenting practice was associated with adolescents’ BMI percentile. Interestingly, monitoring and modeling parenting practices were associated with higher BMI for parents, but they were not associated with adolescents’ BMI percentiles. Further studies that classify the levels of monitoring and modeling parenting practices may help to explain this preliminary finding.

The results of this study also revealed that reasoning and monitoring parenting practices are positively associated with adolescents’ fruit and vegetable consumption. In another study, a warm family environment and enjoyable family meals were shown to be factors for increased fruit and vegetable consumption and lower risk of childhood obesity [35]. Furthermore, the accessibility and availability of fruits and vegetables in the home have been positively associated with family members greater consumption of these foods [36]. Thus, when parents pay attention to different environmental factors, adolescents may increase their consumption of fruits and vegetables.

In addition, a lower consumption of unhealthy snacks among African American adolescents in this study was associated with the four parenting practices of reasoning, monitoring, modeling, and authoritative. This finding is consistent with at least two other studies, showing that the monitoring parenting practices reduce the risk of the consumption of unhealthy snacks, including sweets and sugar-sweetened beverages [37,38]. In a study by Palfreyman et al., three kinds of role modeling were identified: verbal, unintentional, and behavioral modeling [31]. The findings of that study showed that unintentional modeling is related to higher levels of snack consumption [39,40]. However, their findings are not comparable with the results of this study, as the modeling parenting practice was based on both verbal and behavioral modeling. Thus, the type of modeling is important when considering the correlations between modeling and the consumption of a specific food group. Authoritative parenting practices which are warm, protective, and supportive, are positively related to parental attempts to encourage the child to eat fruits, vegetables, and dairy [41]. Two systematic review articles also revealed that children raised with authoritative parenting practices tend to eat healthier foods and have lower obesity rates [27,41]. The findings of this study showed that authoritative parenting practices were associated with lower consumption of unhealthy snacks, which is in agreement with previous findings, showing an increase in healthy habits among adolescents.

The rates of fruit and vegetable consumption have been reported to be less than the recommended level for adolescents [42]. Thus, attention to environmental factors and social support can play a role in adopting healthy eating habits. In this study, a higher consumption of fruits and vegetables and a lower consumption of unhealthy snacks were found in the school environment. This may be a result of school policies, school educational interventions, or the influence of peers. Previous studies have found that school-based interventions not only result in the prevention of excess weight and obesity, but also lead to an increase in the consumption of fruits and vegetables [43,44]. In addition, children attending a school with a school fruit program demonstrate a decreased frequency of unhealthy snack consumption and a higher consumption of fruit instead of unhealthy snacks [45].

Strengths of this exploratory work include participation of parents and youth from five states and the District of Columbia, use of qualitative interviews to assess dietary intake of adolescents, and attention to the research gap regarding identification of parenting practices and associations with dietary behaviors among African American early adolescents.

Limitations include the small sample size; however, associations were observed even with the small sample size. To provide further evidence to support policy changes, another recommendation would be to apply the study design to a larger sample. This work used BMI and BMI-for-age percentile to estimate obesity among parents and adolescents. Although BMI is a widely used measurement, other more precise obesity measurements, such as bio impedence and dual X-ray absorptiometry, which have direct fat/muscle measurements, can provide a more accurate estimation in future studies [46]. According to the demographic data, 86% of adolescents were from single-parent households, which may impact the influence of parenting practices on eating behaviors differently than for adolescents from a co-parenting household. Therefore, control variables, such as household income and single vs. co-parenting household status, need further investigation in the study of how parenting practices influence African American adolescent eating behaviors

## 5. Conclusions

This study focused on the parenting practices of African American dyads and their eating habits. The results of the study indicated that authoritarian parenting practices, which are common among African Americans, may increase the risk of obesity among adolescents. In addition, the four parenting practices of reasoning, monitoring, modeling, and authoritative may encourage a reduction in unhealthy snack consumption, whereas reasoning and monitoring may encourage an increase in fruit and vegetable consumption. Food-related parent–child interaction is a key factor contributing to the healthy habits and well-being of adolescents. The results of this study can be used for improvement in existing nutrition educational interventions and/or the development of new educational interventions for parents in light of cultural differences. Future research may consider subcategories of parenting practices or other essential environmental factors for a profound study of the influence of critical factors on the eating habits and health conditions of African American dyads.

## Figures and Tables

**Table 1:** Food categories consumed by early adolescents aged 10–13 years.

**Dairy category: Dairy ^1^ + dairy sugar-sweetened beverages (SSBs) ^2^**
^1^ Cheese (or foods made with cheese including macaroni and cheese, alfredo, tacos, pizza, sandwich, bean and cheese burrito, egg and cheese bagel); white milk; yogurt; M&M YoCrunch ^2^ Chocolate milk; vanilla strawberry Nesquick; ice cream milk shake
**Fruits and vegetables category: total fruits ^3^ + whole fruits ^4^ + vegetables ^5^**
^3^ Oranges (including mandarin); juice (orange, cranberry, apple, 100% juice Capri-Sun); grapes; pomegranate; mango; Gogo-squeeze applesauce; banana; cantaloupe; apple; pineapple; pears; strawberries ^4^ Orange (including mandarin); grapes; pomegranate; mango; Gogo-Squeeze applesauce; banana; cantaloupe; apple; pineapple; pears; strawberries ^5^ Tomato/marinara sauce (on spaghetti); pizza sauce on pizza; carrots; tomato; beans/legumes (including bean dip); broccoli; lettuce; corn; sub sandwich w/vegetables; potatoes (sweet potatoes, potato salad, mashed); cabbage; okra soup; V8
**Unhealthy food category: sweet ^6^ + salty snack ^7^ + non-dairy SSBs ^8^**
^6^ Sports drinks (Gatorade; Powerade); punch (Hi-C, Pog passion fruit juice, orange drink, Aloe Vera King juice); soda; sweet tea ^7^ Ice cream; sweetened grains (pop-tart, funnel cake, chocolate-covered pretzels, corn bread, muffins, donut, granola bar); candy (M&Ms, Mentos, Lifesavers, Starburst, Twix, Tootsie rolls, sucker, push pop); pie; marshmallows; cookies/bars; brownie; cake; trail mix bar; Fruit by the Foot; fruit-flavored snacks; chocolate syrup; jelly ^8^ Crackers (CheezIt, round butter crackers); pizza bites/rolls/bagels; chips (Pringles, Doritos, barbecue chips, tortilla chips, hot fries, Hot Cheetos, Funyuns, Lay’s Stax); popcorn; pretzels; tater tots

The numbers 1 to 8 represent the food categories and types of foods in each category.

**Table 2:** Items of parent survey to identify parenting practice.

**Monitoring**
How much do you keep track of the sweets (candy, ice cream, cake pastries) that your child eats?
How much do you keep track of the sugary drinks (soda/pop, Kool-Aid) your child drink?
How much do you keep track of the snack foods (potato chips, Doritos, cheese puffs) that your child eats?
How much do you keep track of the high-fat foods (fried foods, french fries) that your child eats?
How much do you keep track of the fruits and vegetables your child eats?
I like to be sure that my child does not eat too many sweets (candy, ice cream, cake or pastries).
I like to be sure that my child does not eat too many high-fat foods.
I intentionally keep some foods out of my child’s reach.
**Reasoning**
How often do you say something positive about the food that your child is eating?
How often do you tell your child how tasty a new food is?
How often do you reason with your child to get him/her to eat (e.g., ‘Milk is good for your health because it will make you strong’)?
How often do you tell your child that healthy food tastes good?
How often do you compliment your child for eating food (e.g., ‘What a good boy! You’re eating your vegetables’)?
How often do you encourage your child to try to eat healthy foods such as vegetables?
I make comments on my eating behaviors/food choices when I am with my child (e.g., ‘I’ll be healthy and have vegetables’).
I try to influence my child’s food preferences by verbally stating my own (e.g., ‘I love carrots, they’re one of my favorites’).
I verbally encourage my child to copy my eating behaviors.
I tend to talk more often about foods I would like my child to eat.
I try to talk more often about foods I would like my child to eat.
I explain my food choices verbally to my child (e.g., ‘I think I’m going to have some fruit, as I like it and it’s good for me’).
**Modeling**
My child has picked up eating behaviors from me which I have not intentionally encouraged him or her to copy (e.g., putting ketchup on most foods, or eating vegetables first).
When I show my child I enjoy fruits and vegetables, he or she tries them.
My child is more likely to try or eat new foods if I eat the new foods with him or her.
My child is more likely to try new foods he or she has seen me eating.
My child asks to try foods from my plate which he or she sees me eating.
How much do you keep track of the milk or foods with calcium, like cheese and yogurt, your child consumes?
How much do you keep track of foods labeled as whole grain that your child eats?
**Copying**
My child has picked up eating behaviors from me which I had tried to hide from him or her (e.g., avoiding certain foods).
My child has copied eating habits from me which I did not realize I had (e.g., salting my food before I taste it).
If I point out certain eating behaviors or foods I like or don’t like, my child is more likely to copy them.
The eating behaviors of other family members influence what my child eats.
My child has picked up eating behaviors from me which I had tried to hide from him or her (e.g., avoiding certain foods).
**Authoritative**
I know exactly when things are not going very well for my child.
When my child is sad, I know what is going on with him or her.
I feel good about the relationship I have with my child.
My child and I have warm affectionate moments together.
I know exactly when my child has difficulty with something.
I find time to talk with my child.
I spend a lot of time with my child.
I easily find a way to make time for my child.
I attend as many of my children’s events and activities as possible.
I find it interesting and educational to be with my child for long periods.
Every free minute I have I spend with my child.
I always help my child with everything he/she does.
**Roles/Expectation**
I expect my child to follow our family rules.
I have clear expectations for how my child should behave.
I have clear expectations for how my child should behave.
I make sure that my child understands what I expect of him or her.
I teach my child to follow rules.
**Authoritarian**
I make sure my child is aware of how much I sacrifice for him or her.
I make my child feel guilty when he or she does not meet my expectations.
When my child hurts my feelings, I stop talking to him/her until he or she pleases me again.
I teach my child to stay in control of his or her feelings at all times.
I do not allow my child to question my decisions.
When I ask my child to do something, I expect him/her to do it immediately without any questions.
I let my child know that I am the boss in our house.
I do not allow my child to get angry with me.
When my child has lost something, I stop what I am doing to find it before he/she gets too upset.
I do not let my child get involved in activities or tasks where he/she may potentially fail.
**Neglecting**
I have a hard time consistently enforcing rules with my child.
I do not always follow through when I threaten to discipline my child.
I threaten discipline more often than I actually give it.
When I discipline my child, I sometimes end the punishment early.
There are times I just do not have energy to make my child behave as he or she should.
When my child does something that is not allowed, I do not talk to him or her until he or she says he or she is sorry.
I am less friendly with my child if he or she does not see things my way.

**Table 3:** Factor analysis for parenting practices.

Parenting Practice	# of Items	Factor Loadings (Min–Max)	% of Variance Explained	Mean (SD)	Median (IQR)
Reasoning	8	0.42–0.83	5.70%	3.59 (1.01)	4.0 (3.0–4.4)
Monitoring	12	0.45–0.85	5.30%	3.66 (1.05)	3.7 (3.1–4.5)
Modeling	7	0.44–0.82	4.10%	3.72 (1.22)	4.0 (3.6–4.4)
Copying	4	0.60–0.88	3.30%	3.59 (1.44)	4.1 (2.0–4.8)
Authoritative	12	0.49–0.83	7.40%	4.41 (0.95)	4.8 (4.4–4.9)
Setting rules	5	0.78–0.87	5.10%	4.56 (1.07)	5.0 (4.4–5.0)
Authoritarian	10	0.43–0.84	4.90%	3.36 (0.91)	3.2 (2.7–3.8)
Neglecting	7	0.47–0.67	3.70%	2.78 (1.09)	2.6 (1.7–3.3)

# = number. It is the number of items for each parenting practice category.

**Table 4:** Demographic data of the sample.

Variables	Number	Percentage
Adolescent Age (years)		
10	12	85.71
13	2	14.29
Adolescents Sex		
Male	6	42.86
Female	8	57.14
Parent Age (years)		
26–34	9	64.29
35–54	5	35.71
Parent Sex		
Male	3	21.43
Female	11	78.57
Parents Education		
Below high school	1	7.14
Diploma or GED	3	21.43
Some college or technical school	8	57.14
Four-year college and above	2	14.29
Household Income (USD)		
Below 25,000	7	50
25,000–44,999	7	50
Marital Status		
Single	9	64.29
Married	2	14.29
Separated	1	7.14
Divorced	2	14.29
Location		
Georgia	4	28.57
Hawaii	1	7.14
Minnesota	1	7.14
Ohio	1	7.14
Connecticut	2	14.29
District of Columbia	5	35.71

**Table 5:** Correlation between parenting practices and obesity.

Parenting Practice	Adolescent BMI Percentile		Parent BMI	
	Correlation	*p*-Value	Correlation	*p*-Value
Reasoning	−0.16	0.60	0.48	0.23
Monitoring	−0.08	0.79	0.67	0.07
Modeling	0.10	0.73	0.89	<0.01
Copying	0.38	0.19	0.02	0.95
Authoritative	0.15	0.63	−0.01	0.98
Setting rules	0.06	0.84	−0.26	0.52
Authoritarian	0.50	0.08	−0.07	0.87
Neglecting	0.07	0.81	0.45	0.26

**Table 6:** Correlation between parenting practices and number of eating occasions by food category.

Parenting	Dairy		Fruits and Vegetables		Unhealthy Snacks	
Practice	Correlation	*p*-Value	Correlation	*p*-Spearman	Correlation	*p*-Spearman
Reasoning	0.28	0.34	0.60	0.02	−0.69	<0.01
Monitoring	0.28	0.34	0.63	0.02	−0.72	<0.01
Modeling	0.05	0.87	0.38	0.19	−0.56	0.04
Copying	0.18	0.54	0.03	0.91	−0.11	0.71
Authoritative	0.37	0.20	0.36	0.20	−0.58	0.03
Setting rules	−0.10	0.76	−0.13	0.65	−0.02	0.96
Authoritarian	0.23	0.43	0.01	0.98	0.12	0.68
Neglecting	−0.11	0.71	−0.05	0.86	−0.14	0.62

**Table 7:** Environmental factors and eating occasions of foods.

	Total EOs	Dairy		Fruits and Vegetables		Unhealthy Snacks	
	*n* (%)	*n* (%)	*p*-Value	*n* (%)	*p*-Value	*n* (%)	*p*-Value
EO location			0.89		0.01		0.05
Home	57 (61.3)	15 (73.1)		19 (45.2)		22 (61.5)	
Car/bus	8 (8.6)	1 (3.8)		1 (3.2)		6 (17.9)	
School	19 (20.4)	4 (15.4)		13 (46.8)		3 (7.7)	
Others	9 (9.7)	2 (7.7)		2 (4.8)		5 (12.8)	
EO activity			0.98		0.09		0.21
Just eating	34 (36.6)	8 (34.6)		9 (29.0)		16 (46.2)	
Performing an activity	59 (63.4)	14 (65.4)		26 (71.0)		20 (53.8)	
EO accompany			0.98		0.8		0.85
Non-IEOs	76 (81.7)	18 (81.0)		29 (82.0)		29 (80.0)	
IEOs	17 (18.3)	4 (19.0)		6 (18.0)		7 (20.0)	

## Data Availability

Data used during the current study are available from the corresponding author.
